# The Effects of Mental Imagery with Video-Modeling on Self-Efficacy and Maximal Front Squat Ability

**DOI:** 10.3390/sports4020023

**Published:** 2016-04-14

**Authors:** Daniel J. M. Buck, Jasmin C. Hutchinson, Christa R. Winter, Brian A. Thompson

**Affiliations:** Springfield College, Massachusetts, MA 01109, USA; dbuck@springfieldcollege.edu (D.J.M.B.); cwinter@springfieldcollege.edu (C.R.W.); bthompson@springfieldcollege.edu (B.A.T.)

**Keywords:** strength and conditioning, mental skills, psychology, three repetition maximum (3RM)

## Abstract

This study was designed to assess the effectiveness of mental imagery supplemented with video-modeling on self-efficacy and front squat strength (three repetition maximum; 3RM). Subjects (13 male, 7 female) who had at least 6 months of front squat experience were assigned to either an experimental (*n* = 10) or a control (*n* = 10) group. Subjects′ 3RM and self-efficacy for the 3RM were measured at baseline. Following this, subjects in the experimental group followed a structured imagery protocol, incorporating video recordings of both their own 3RM performance and a model lifter with excellent technique, twice a day for three days. Subjects in the control group spent the same amount of time viewing a placebo video. Following three days with no physical training, measurements of front squat 3RM and self-efficacy for the 3RM were repeated. Subjects in the experimental group increased in self-efficacy following the intervention, and showed greater 3RM improvement than those in the control group. Self-efficacy was found to significantly mediate the relationship between imagery and front squat 3RM. These findings point to the importance of mental skills training for the enhancement of self-efficacy and front squat performance.

## 1. Introduction

The front squat, which is used extensively in strength training programs at the collegiate level and higher [1], is one of the few exercises which can develop the majority of the lower body musculature as well as increase abdominal and lower back strength and stability [2]. Strength training programs are often created based on percentages of a one repetition maximum (1RM) in order to train for the desired results (e.g., hypertrophy, strength, power). The front squat is instrumental for all three aspects of training. Given the prevalence and importance of the front squat exercise in strength training programs, providing a method of increasing the load lifted during the exercise seems valuable. Increasing an athlete**′**s 1RM shows an improvement in strength, and in the case of the squat, indicates an increased ability to produce force into the ground, increasing attributes such as vertical jump and acceleration for the enhancement of sports performance (e.g., [3]). The front squat is also a vital component of the clean, an important Olympic lift for the development of power, as the clean requires the ability to catch the bar on the shoulders in the same position as one front squats from. Achieving proper depth is also paramount when performing the clean with greater loads on the bar, as it requires the performer to drop down quickly and lower their body under the bar to catch the weight [4]. One often overlooked aspect in strength development is the use of psychological strategies, particularly more complex techniques such as mental imagery [5].

### 1.1. Mental Imagery

Mental imagery, defined as a cognitive process during which people use their minds to create (or recreate) experiences that are similar to real-life situations [6] has been found to enhance performance in a variety of sport settings [7]. However, the use of imagery during weight training has not been studied in great depth, to date. In early experimental studies, mental imagery was largely shown to be ineffective in enhancing the performance of strength-based tasks [8]. A meta-analysis conducted by Feltz and Landers [9] found a relatively small effect size (Cohen**′**s d = 20) for the effects of imagery on strength tasks. However, Wright and Smith [10] point out that the majority of these studies used a traditional “visualization” imagery approach, (*i.e.*, focusing on the visual aspects of imagery) in contrast to a more comprehensive (physical, response-based) imagery approach. They argue that the “lack of functional equivalence afforded by these ‘visualization**’**-type interventions may, therefore, explain their ineffectiveness”.

Based upon the principle that imagery enhances performance because the same neurophysiological processes underlie imagery and actual movement [11,12], Holmes and Collins [13] developed the PETTLEP model. PETTLEP is an acronym for the proposed key elements to an effective imagery intervention (*i.e.*, physical, environment, task, timing, learning, emotion, and perspective components); this approach aims to maximize functional equivalence by ensuring that the imagery performed is a close representation of the actual movement. Wright and Smith [10] demonstrated the superiority of PETTLEP imagery supplemented with video over traditional (visualization) imagery in a strength task (bicep curl 1RM). In evaluating the results of their study, Wright and Smith highlighted the need for future research on the strength training effects of the PETTLEP model, and suggested that future studies focus on muscles of different sizes and on the PETTLEP imagery effects over different periods of time.

### 1.2. Modeling

Modeling, or observational learning, has been defined as a process in which the performer attempts to imitate an observed action or skill performed by another individual [14]. Like imagery, modeling is a cognitive process by which people can learn a variety of skills and behaviors [15]. Indeed, athletes frequently use modeling for motor skill acquisition and execution [16]. It is theorized that in doing so, an athlete symbolically encodes information about the skill as the demonstration is observed (*i.e.*, they create an internal cognitive representation of the skill), and then uses this encoded information as a guide for future action [17]. It is important to note that this need not be a deliberate process. Cross *et al.* [18], for example, provide evidence of similar neural representations for physically rehearsed and passively observed movement sequences.

Research has typically addressed modeling and imagery as separate and distinct processes, however several investigators [9,19,20] have noted that modeling and imagery are similar; both involving the use of cognitive representations and rehearsal prior to the actual physical execution of the skill [17]. During modeling, information about the skill is encoded into a cognitive representation, while during imagery a cognitive representation or image is recalled from memory; these mechanisms have been shown to have similar neural involvement [21]. While the distinction between modeling and imagery is important from an academic perspective, the effects of a combined intervention may be of greater practical interest to coaches and practitioners. Indeed, it has been suggested that, in order to optimize functional equivalence, performers can make use of pictures, sound, and/or video clips to provide stimulus and response information for imagery [22]. McCullagh, Law and Ste-Marie [23] have recently advocated for research on imagery interventions that include modeling as a component.

### 1.3. Self-Efficacy

Self-efficacy (SE), which is defined by Bandura [24] as “beliefs in one′s capabilities to organize and execute the courses of action required to produce given attainments”, is derived from four sources of information; mastery experiences (*i.e.*, past performances), vicarious experiences, verbal persuasion, and physiological states. Compared with persons who doubt their capabilities, those with high SE for accomplishing a task participate more readily, set more challenging goals for themselves, persist longer in the face of adversity, and achieve at a higher level [24]. Previous research in a strength setting has examined the effect of perceptions of goal difficulty on lower body strength performance. Participants who were misinformed during a 3RM deadlift task (*i.e.*, were unknowingly lifting 10% above their actual 3RM to create lower perceived demand), lifted a signficantly heavier load compared to participants who received correct lift information (high perceived demand) [25]. This finding highlights potential SE barriers to performance success.

Modeling and imagery are classified as similar processes within SE theory; both can serve as vicarious experiences and provide information that affects SE [17]. Imagery itself can also serve as a source of SE, as enactive mastery experience can be attained through the use of imagery [24]. Bandura has placed much emphasis on the mediating role that SE plays in the observation-behavior change relationship [24]. To date, little research has examined this mediating effect, but that which has is largely supportive of Bandura**′**s theoretical proposition. For example, using hierarchical multiple regression analyses, Beauchamp *et al.* [26] demonstrated that pre-competition imagery use accounted for significant variance in both SE and performance in collegiate golfers. Moreover, SE was predictive of golf performance, and imagery use mediated the relationship between SE and performance. The mediating role of SE, in regards to the effects of imagery, has yet to be examined in a strength setting.

The current study was primarily designed to determine if mental imagery (using the PETTLEP model) supplemented with video-modeling, would be effective in increasing front squat 3RM. Self-efficacy was measured in order to probe the underlying mechanism by which imagery may have an effect on 3RM; that is to determine if imagery effects on front squat were mediated by SE. It was hypothesized that (1) imagery would significantly enhance SE for the front squat; (2) imagery would significantly enhance front squat 3RM; and (3) the effect of imagery on front squat 3RM would be mediated by changes in SE for the 3RM. The effect on front squat was expected to be small, given the upper limits of performance, but it is noted that even a slight trend towards a greater 3RM may still be of practical importance.

## 2. Materials and Methods

### 2.1. Experimental Approach to the Problem

The objective of this study was to determine the effects of mental imagery supplemented with video-modeling on SE and performance during the front squat exercise. Subjects were assigned to either an experimental (*i.e.*, mental imagery supplemented with video-modeling) group or a control (placebo video) group. Pre- and post-intervention measurements of front squat 3RM (lbs.) and SE for the 3RM were collected and compared using two 2 (group) × 2 (time) mixed factorial analyses of variance (ANOVAs).

### 2.2. Determination of Sample Size

A power analysis was conducted, using G*Power 3.1 [27], to establish appropriate sample size for a 2 (Group) × 2 (Time) mixed-factorial ANOVA. Alpha was set at 0.05 and power at 0.8 to protect beta at four times the level of alpha [28]. Predicting a moderate size effect (*f* = 0.28) of the intervention on the dependent variables [10], it was estimated that 20 subjects would be required.

### 2.3. Subjects

Twenty subjects (13 male, 7 female; age, 22.67 ± 1.79 years) were recruited and placed into one of two groups (experimental or control) in an alternating fashion by gender, based on the order in which each subject was recruited. There were a total of 10 subjects in the treatment group and 10 in the control group. None had any prior experience using imagery deliberately for the purpose of rehearing or improving performance on an athletic task. Inclusion criteria were that all subjects had at least 6 months of experience with the front squat exercise (*i.e.*, intermediate training status [29]) and had no recent injuries that would prevent them from effectively performing the front squat exercise. The study was approved by the Institutional Review Board, and all subjects provided written informed consent prior to testing.

### 2.4. Procedures

Two days of testing were required for this study. To allow for complete recovery of skeletal muscle contractility [30] and to provide adequate time for subjects to practice the imagery protocol testing sessions were separated by three days. Prior to the first test subjects were asked to refrain from any training involving the legs for two days and to refrain from all training for 24 h prior to testing. No exercise was allowed between testing sessions. The subjects were also asked to consume a similar diet before each testing session and to refrain from caffeine consumption for 6 h prior to testing. Testing sessions for each individual were conducted at the same time of day to control for any potential diurnal variation. Each session began with self-myofascial release using a foam roller followed by a series of activation exercises and static and dynamic stretches (see supplementary materials for details).

Subjects performed the front squat using a Keiser rack (Knoxville, TN, USA) and York barbell (York, PA, USA). The subject′s foot position, grip width, and grip style were self-selected (all elected to use a clean grip). A 3RM test was chosen over a 1 for reasons of safety [31]. Each subject started the warm-up 5 × 60% of estimated 3RM (estimation based on recent training logs) and then progressed through the following sets: 4 × 70% estimated 3RM, 3 × 80% estimated 3RM, and 2 × 90% estimated 3RM. After these warm up sets were completed, a 3 × 100% attempt was made. If successful, the weight was increased by 10–20 lbs. per subsequent attempt until their 3RM was determined. The intervention for the experimental group consisted of a video-enhanced imagery protocol. Each subject was recorded using a Samsung Schneider Kreuznach Camcorder (Taegu, Korea) during their 3RM front squat on Day 1. Recording took place slightly forward of the subject′s right side for the entire three repetitions in order to be able to determine proper squat depth (*i.e.*, femur parallel to the ground). If the subject was able to successfully complete a subsequent 3RM with a greater load, only the highest successful 3RM video was saved.

Following completion of the 3RM protocol, subjects viewed their own video, and were provided with a corresponding imagery script. Subjects were then sent an e-mail with a copy of their video as well as a video of one of two individuals (one male, one female) completing the front squat with excellent technique. As perceived similarity to models is an important attribute for maximizing SE [24], the models were the same age range as the subjects, and subjects were each sent a video-model matching their identified gender. Subjects were asked to view both videos six times between testing sessions (twice each day), and to use the script provided to image themselves performing the front squat with the same technique as the model lifter. Reminder texts were sent to the subjects once each day between testing days in order to remind them to watch the videos. The imagery script followed PETTLEP guidelines; subjects were directed to visualize the front squat in their usual lifting environment and to visualize the movement in real time, paying particular attention to the technical aspects of the movement (e.g., head, spine and hip alignment) using the model as a guide. They were also directed to exert emotional control and to feel their muscles activate as if they were physically performing the exercise. Subjects kept an imagery log in which they recorded the time and date of their imagery sessions; this log was checked prior to the second session to ensure adherence to the protocol. The control group received a placebo video, a video of a baby squatting, which they were asked to view the same number of times (*i.e.*, twice a day for three days) as the experimental group. This video was intended to parallel the videos seen by the experimental group by providing similar visual material, but no usable information.

On Day 2, following all warm-up procedures and prior to beginning the testing set, experimental subjects viewed the model video as well as their own 3RM video from Day 1. After viewing the videos, subjects were guided through the imagery protocol by the researcher. Particular emphasis was placed on kinesthetic elements and imagining the lift at the speed at which it would occur. Subjects were told that they may view either video again prior to the testing set if they desired (two subjects elected to do so). Control group subjects viewed the placebo video prior to beginning the testing set.

Subjects′ SE for the 3RM was assessed immediately prior to each testing set using a task-specific SE scale, which was constructed according to Bandura′s guidelines [32]. Bandura asserts that “scales of perceived self-efficacy must be tailored to the particular domain of functioning that is the object of interest”, therefore subjects were asked to rate their confidence in their ability to perform the front squat with maximal weight (*i.e.*, the weight currently being attempted) and proper technique on a scale ranging from 0 (cannot do at all) to 100 (highly certain I can do). If a subsequent set of three repetitions was completed successfully, this measurement process was repeated. Only the SE data associated with the highest successful 3RM was saved and used for data analysis.

### 2.5. Statistical Analysis

Two 2 × 2 mixed factorial ANOVAs were conducted to analyze the SE and 3RM data; the independent groups factor was group (experimental/control) and the repeated measures factor was time (pre/post). The significance level for each ANOVA was set at 0.05. Additional post-hoc tests were conducted using independent as well as paired samples *t*-tests; the Bonferroni adjusted significance level for the *t*-tests was set at 0.00625. Cohen′s effect size *d* was also computed for all post hoc comparisons relying on pooled standard deviations when appropriate.

Where interaction effects were significant, regression analyses [33] were conducted to investigate the hypothesis that SE mediates the effect of mental imagery on 3RM performance. To facilitate this analysis, change scores (*i.e.*, pre intervention—post intervention) were calculated for SE and 3RM. The first step of the analysis was to regress the dependent variable (3RM change) on the independent variable (group) in order to confirm that the independent variable was a significant predictor of the dependent variable. Following this the mediator (SE change) was regressed on the independent variable (group) to confirm that the independent variable was a significant predictor of the mediator. Finally, the dependent variable (3RM change) was regressed on both the mediator (SE change) and independent variable (group) in order to confirm that the mediator was a significant predictor of the dependent variable, while controlling for the independent variable. IBM SPSS version 21 was used for data analysis.

## 3. Results

**T**he current study was designed to determine if mental imagery supplemented with video-modeling was effective in increasing self-efficacy and 3RM in the front squat. Twenty subjects (*n*_exp_ = 10 and *n*_ctrl_ = 10) completed the study. 3RM tests were separated by 3 days to allow for full recovery and to provide time for subjects to practice the imagery protocol. Descriptive statistics for the dependent variables are presented in Table 1.

Analyzing the SE data, a significant group x time interaction was revealed, *F*(1, 18) = 6.65, *p* = 0.019, η^2^ = 0.27. Follow-up post-hoc tests indicated no significant difference in SE scores between groups pre intervention, *t*(18) = 0.27, *p* = 0.792, *d* = 0.12; however, a significant difference was determined post intervention with the experimental group reporting higher SE scores, *t*(18) = 3.08, *p* = 0.006; *d* = 1.40. In addition, no significant difference in SE scores of the control group was found from pretest to posttest, *t*(9) = 1.26, *p* = 0.238, *d* = 0.40, while the SE scores of the experimental group increased significantly from pretest to posttest, *t*(9) = −4.71, *p* = 0.001, *d* = −1.49.

The analysis of the 3RM strength data also revealed a significant group by time interaction, *F*(1,18) = 5.73, *p* = 0.028, η^2^ = 0.24. Follow-up post-hoc tests indicated no significant difference in 3RM strength between groups pre, *t*(18) = 0.18, *p* = 0.862, *d* = 0.08, and post intervention, *t*(18) = 0.43, *p* = 0.669, *d* = 0.20. The experimental group, however, showed a significant increase (Mdiff = 8.5 lbs.) in 3RM strength from pretest to posttest, *t*(9) = −7.97, *p* = .000, *d* = −2.52. The pre- to posttest increase for the control group (Mdiff = 2.5 lbs.) was not significant, *t*(9) = −1.10, *p* = 0.299, *d* = −0.35.

Given the significant group by time interactions for both dependent variables, linear regression was used to investigate the hypothesis that SE mediates the effect of video-supplemented mental imagery on 3RM performance. Regression results indicated that Group was a significant predictor of SE change, β = 0.532, *p* = 0.016, and that SE change was a significant predictor of 3RM change, β = 0.654, *p* = 0.002. These results support the mediational hypothesis. Experimental group initially predicted 3RM change (step one of the analysis; β = 0.492, *p* = 0.028), but was not a significant predictor of 3RM change after controlling for change in SE, β = 0.250, *p* = 0.257, consistent with partial mediation. Approximately 46% of the variance in 3RM change was accounted for by change in SE (*R*^2^ = 0.457).

## 4. Discussions

The results of this study indicate that a mental imagery protocol supplemented with video-modeling is one mechanism by which SE might positively influence front squat 3RM.

Subjects who followed the video-imagery protocol showed a 10.7% increase in SE while subjects in the control group decreased in SE by a similar amount (12.3%). It is unknown why SE would decrease in the control group. It could be speculated that, without any meaningful intervention, the control subjects may have lacked motivation to repeat the task in such a short time-frame. Given the proposed bidirectional relationship between SE and motivation [24], this could account for the decline in SE.

Overall, our findings are in agreement with Silbernagel *et al.* [9] who found a positive association between imagery use and SE. This notion is of importance to strength coaches because SE is significantly and positively related to athletic performance [34,35]. Moreover, previous research has shown SE to have greater influence on performance in non-continuous sport conditions (consisting of discrete trials) when compared to continuous, dynamic sports [36], suggesting that strength and conditioning settings may be optimal for implementing this type of intervention. Future research involving elements of strength training other than 3RM would be valuable.

It has previously been reported [6] that athletes who were more confident in their ability to use imagery used it more often, creating a positive cycle of imagery use and SE. Although pre-task imagery use was found to influence SE and performance in the present study, future research should consider the theorized bidirectional relationship between SE and imagery use and examine the interplay of these variables in greater depth.

According to SE theory [14,24], enhanced SE should lead athletes “to set higher goals, exert more effort and persist resiliently in the face of adversity, all of which are likely to contribute to improved performances” [26]. In this study, there was a significantly greater increase in 3RM for the experimental group than for the control group. These findings are in line with those previously reported by Wright and Smith [10] who observed superior strength gains following a video-supplemented PETTLEP imagery program compared to other conditions (*i.e.*, traditional imagery, physical practice, and control). Thus, the results of the study provide additional empirical support for the use of comprehensive, video-supplemented imagery in strength tasks. This study extends previous findings [11,12] by demonstrating the mediating role of SE in the imagery-performance relationship in a strength task.

While the results of the present study are encouraging, some methodological limitations should be noted. The completed imagery logs suggest that participants attempted to complete the intervention task as instructed, but without a direct measure of imagery success we can only infer that they indeed used an imagery strategy. It is possible that the experimental subjects used a declarative observational strategy instead of (or in addition to) imagery, wherein they made visual comparisons between their performance and that of the model and subsequently changed certain aspects of their technique to match the model lifter. Further, despite the use of a control group, it is not possible to rule out potential effects of subject expectations. While neither group was explicitly advised that the intervention was designed to improve performance, it is more likely that the experimental group considered that the intervention might improve their 3RM. Perhaps a more effective control, for future studies seeking to replicate these findings, might be to use the same videos as for the experimental group but assign a different task (one that precludes the explicit use of imagery; e.g., making verbal descriptions of what they see).

No attempt was made to control for individual differences in modelling or imagery ability. As with any skill, imagery may be more effective for some individuals than others, depending on their ability to utilize imagery effectively [35]. Future studies might be conducted over a longer time period in order to develop imagery skills before posttest data are recorded. Such research may also be beneficial to better understand how imagery affects maximal lifting over time, as subtle changes in technique may require more time to translate into improved 3RM scores. Bandura advocates that gradations of challenge [32] be built into efficacy scales. In the present study we adopted the staggered approach described as 3RM values were not known at the first trial. Studies taking a more longitudinal approach could make use of a graded SE scale, specific to each individual′s previous 3RM (*i.e.*, subjects′ efficacy to achieve a 3RM that is 3%, 6%, 9%, *etc*. greater than before). A baseline trial to establish 3RM could also be used to achieve this.

Subjects′ current athletic status or background was not considered in the current study, thus a comparison of competitive vs. recreational athletes may be a worthwhile future endeavor. It might also be interesting to explore the effects of mental imagery with video-modeling in less-trained populations, particularly as neuroimaging studies have shown that the neural networks activated by mental imagery differ between novices and experts [37]. Another valuable extension of the present study might be to couple mental imagery with movement sequences (*i.e.*, dynamic imagery). Recent research indicates dynamic imagery may be superior in terms of enhancing technical efficacy and temporal congruence between imagery and performance [38].

The present study did not separate the effects of self- and other-modeling. Thus, it is unclear whether vicarious experience (viewing others) or personal accomplishment (viewing self) combined with mental imagery was more enhancing to SE and/or 3RM. Future research that endeavors to disentangle these effects is warranted and may further our understanding of the effects of imagery and video-modeling in a strength and conditioning setting.

## 5. Conclusions

The use of mental imagery with video-modeling appears to be an effective intervention to enhance SE for the front squat and front squat 3RM. Specifically, the current study demonstrates that front squat SE and 3RM are significantly (*p* < 0.001) increased following a brief (3-day) video-supplemented imagery intervention. As far as a contribution to theory, the results of the mediational analysis predicting performance are consistent with theorizing by Bandura [24] and point to mental imagery with video-modeling as a factor through which SE influences performance.

The application of mental skills, such as imagery, in the strength and conditioning field is growing but remains under-utilized at present [5]. Mental skills are an important aspect of athletic development and, like physical skills, should be practiced often and with purpose. One benefit to mental imagery is that, once learned, it can be practiced anywhere at any time, moreover recent advances in technology have made digital recording more accessible than ever before and video can be easily distributed to athletes to supplement their mental skills training.

Video-supplemented imagery may be particularly useful for athletes who have difficulty in generating, maintaining, and controlling mental images. For college/university strength coaches who generally have athletes over a 4 to 5 year span, the opportunity exists to develop a comprehensive program of mental imagery supplemented with video-modeling which may be very helpful for assisting in athlete learning and development, potentially leading to improved SE and performance in the weight room as well as in athletic competition. In sum, mental imagery should be considered as a part of strength and conditioning programs with a goal to improve athletic performance and team success.

## Figures and Tables

**Table 1:** Descriptive statistics and effect sizes for experimental and control groups.

Variable	Pretest		Posttest		Cohen’s *d*
	Mean	*SD*	Mean	*SD*	
3RM (lbs.)					
Experimental	183.00	61.92	191.50	62.41	−2.52
Control	179.00	36.04	181.50	37.57	−0.35
Self-Efficacy					
Experimental	82.40	6.75	90.10	7.85	−1.49
Control	83.50	11.10	76.70	11.27	+0.40

## Supplementary Files

Supplementary File 1

## Abbreviations

The following abbreviations are used in this manuscript:

3RM	Three repetition maximum
1RM	One repetition maximum
PETTLEP	Physical, environment, task, timing, learning, emotion, and perspective components (elements of an effective imagery intervention)
SE	Self-efficacy
