# Supplementary material for: The Effects of Mental Imagery with Video-Modeling on Self-Efficacy and Maximal Front Squat Ability
Source: Sports (Basel). 2016 Apr 14;4(2):23. doi: 10.3390/sports4020023 (PMC5968921; doi:10.3390/sports4020023)
Supplement: Supplementary File 1 [file sports-04-00023-s001.pdf]

Supplementary Materials: The Effects of Mental Imagery with Video-Modeling on Self-Efficacy and Maximal Front Squat Ability

Daniel J. M. Buck, Jasmin C. Hutchinson, Christa R. Winter and Brian A. Thompson

Warm-Up Protocol

Self Myofascial Release (20 s each)

- Calves
- Quadriceps
- Hamstrings
- Gluteals

Static Stretching (20 s each)

- Quadriceps
- Hamstring
- Gluteals

Activation & Supplemental

- Alternate Leg Lower × 5 each leg
- Glute Bridge March × 5 each leg
- Cook Squat × 5

Dynamic (15 yards)

- Knee Hug Lunge
- Quad Walk w/Reach
- Figure 4
- Spiderman Walk
- RDL Walk
- Straight Leg Kick

© 2016 by the authors; licensee MDPI, Basel, Switzerland. This article is an open access article distributed under the terms and conditions of the Creative Commons by Attribution (CC-BY) license (http://creativecommons.org/licenses/by/4.0/).
